# The associations of birth intervals with small-for-gestational-age, preterm, and neonatal and infant mortality: a meta-analysis

**DOI:** 10.1186/1471-2458-13-S3-S3

**Published:** 2013-09-17

**Authors:** Naoko Kozuki, Anne CC Lee, Mariangela F Silveira, Cesar G Victora, Linda Adair, Jean Humphrey, Robert Ntozini, Robert E Black, Joanne Katz

**Affiliations:** 1 Department of International Health, Johns Hopkins Bloomberg School of Public Health, 615 N. Wolfe St., Baltimore, MD 21205, USA; 2 Brigham and Women’s Hospital, 75 Francis Street, Boston, MA 02115, USA; 3 Programa de Pós-graduacao em Epidemiologia, Universidade Federal de Pelotas, Rua Marechal Deodoro 1160, 3o piso, Centro, CEP 96020-220, Pelotas, RS, Brazil; 4 University of North Carolina School of Public Health, 135 Dauer Drive, Chapel Hill, NC 27599, USA; 5 Zvitambo, No 1 Borrowdale Road, Borrowdale, Harare, Zimbabwe

## Abstract

**Background:**

Short and long birth intervals have previously been linked to adverse neonatal outcomes. However, much of the existing literature uses cross-sectional studies, from which deriving causal inference is complex. We examine the association between short/long birth intervals and adverse neonatal outcomes by calculating and meta-analyzing associations using original data from cohort studies conducted in low-and middle-income countries (LMIC).

**Methods:**

We identified five cohort studies. Adjusted odds ratios (aOR) were calculated for each study, with birth interval as the exposure and small-for-gestational-age (SGA) and/or preterm birth, and neonatal and infant mortality as outcomes. The associations were controlled for potential confounders and meta-analyzed.

**Results:**

Birth interval of shorter than 18 months had statistically significant increased odds of SGA (pooled aOR: 1.51, 95% CI: 1.31-1.75), preterm (pooled aOR: 1.58, 95% CI: 1.19-2.10) and infant mortality (pooled aOR: 1.83, 95% CI: 1.19-2.81) after controlling for potential confounding factors (reference 36-<60 months). It was also significantly associated with term-SGA, preterm-appropriate-for-gestational-age, and preterm-SGA. Birth interval over 60 months had increased risk of SGA (pooled aOR: 1.22, 95% CI: 1.07-1.39) and term-SGA (pooled aOR: 1.14, 95% CI: 1.03-1.27), but was not associated with other outcomes.

**Conclusions:**

Birth intervals shorter than 18 months are significantly associated with SGA, preterm birth and death in the first year of life. Lack of access to family planning interventions thus contributes to the burden of adverse birth outcomes and infant mortality in LMICs. Programs and policies must assess ways to provide equitable access to reproductive health interventions to mothers before or soon after delivering a child, but also address underlying socioeconomic factors that may modify and worsen the effect of short intervals.

## Introduction

Providing access to family planning in low- and middle-income countries (LMIC) has social and economic benefits, but is also a critical public health intervention that may increase survival and improve health of mothers and newborns. Short and long birth intervals, or the time between previous and index live births, have been linked to adverse neonatal outcomes, including child mortality, low birthweight, preterm birth, and intrauterine growth restriction (IUGR) [1-3]. Studies have reported J-shaped risk associations, with the highest risk occurring for children born after the shortest birth intervals, then dropping to a plateau roughly around 36 months, then a gradual increase beginning around 60 months [1,3]. Better quantifying the magnitude of these associations may provide invaluable information to estimate the possible impact of family planning interventions in reducing adverse birth outcomes, and the potential for saving newborn lives and reducing stillbirths.

Several mechanisms have been proposed linking short birth intervals with adverse pregnancy outcomes, which were recently systematically reviewed by Conde-Agudelo et al [4]. The maternal depletion syndrome (MDS) postulates that a mother may not be physiologically recovered from the previous birth if she conceives the next child shortly thereafter, leading to adverse outcomes [5,6]. Alternately, short birth intervals may simply be an indicator of non-biological mechanisms. The sibling competition theory hypothesizes that too many children shortly spaced may place resource pressures on families. Short intervals may also be a result of the mother suboptimally breastfeeding the previous child, as proper breastfeeding delays the mother regaining fecundity. Finally, a mother may have a history of preterm births, making the short interval its product rather than preterm birth a product of the short interval.

Long birth intervals, whether intended or unintended, may also have negative outcomes, and thus important to understand the associations. A woman’s physiologic and anatomic capacity to accommodate fetal growth may revert to a nulliparous state if she has undergone a long period since her last birth, and that the infant subsequent to a long interval may experience the same risks as a first birth [7]. The long interval may also be correlated with negative outcomes if it is not a result of conscious family planning; for instance, mothers may be struggling with secondary infertility.

There are limitations to the current literature on this topic. Much of the literature on this subject utilizes cross-sectional studies such as Demographic and Health Surveys (DHS), especially with long recall periods [1-3,8]. Causal inference is difficult to draw from cross-sectional studies, and the quality of both exposure and outcome measures may be poor in datasets that heavily depend on maternal recall. Another major drawback to synthesizing the current evidence is the substantial heterogeneity in definitions of exposures and outcomes across studies.

Thus, the aim of this work is to address some of these limitations by examining the association between birth intervals and poor neonatal outcomes (small-for-gestational-age (SGA), preterm, neonatal and infant mortality), using original data from prospective birth cohort studies conducted in LMIC, and conducting analyses using standardized categorizations and definitions of risk exposure and outcome variables. We controlled for available socioeconomic, nutritional, and reproductive health confounders in each dataset. The ultimate objective is to generate estimates to feed into the Lives Saved Tool (*LiST*). *LiST* is a computer-based tool that estimates the impact of scaling up various health interventions, such as family planning, on maternal and child mortality [9]. This work was conducted to make recommendations regarding the link connecting contraceptive use to adverse neonatal outcomes.

## Methods

### Identification and description of studies included in the analysis

For this analysis, we identified individual prospective birth cohorts from LMICs, conducted a standardized set of primary analyses to answer the study objectives, and performed meta-analyses to derive pooled effect sizes. The birth cohorts were identified from a separate activity assessing the association between SGA/preterm and neonatal and infant mortality [10].

Briefly, we conducted a literature review in February 2009 to identify prospective birth cohorts from LMICs that had data available on gestational age, birthweight, and vital status systematically collected through at least the neonatal period (28 days). Medline, WHO regional database, and bibliographies of key articles were searched, and additionally, Child Health Epidemiology Reference Group investigators were also consulted to identify potential datasets by word-of-mouth. Investigators with birth cohorts containing the minimum required data were contacted to conduct a standard set of analyses or to contribute their data to the analyses. More details on the search strategy are available elsewhere [10]. For inclusion in the present analysis, datasets also had to include data on birth interval, and parity and maternal age information as control variables.

In total, five birth cohort datasets from three countries (Brazil, Philippines, Zimbabwe) were included [11-15], totaling 32,670 singleton live births, of which 19,240 had relevant information. Initial year of data collection in these studies ranged from 1982 to 2004. All studies were conducted in urban locations, and four of the five studies were facility-based. One study was a randomized controlled trial, while the rest were longitudinal surveys. Gestational age was collected using different methodologies: the Brazil studies used date of last menstrual period (LMP) (1982), LMP and the Dubowitz method [16] (1993), and LMP, the Dubowitz method, and ultrasound (2004), the Philippines study used LMP and Ballard method [17], and the Zimbabwe study used the Cappuro method [18]. In the Zimbabwe study, mother-child pairs were enrolled within 96 hours of delivery, while the other studies enrolled prior to or at birth. See Table 1 for further descriptions of the original cohorts.

**Table 1:** Description of studies included in the analysis

				Data from full original cohort, including those not retained in the birth interval analysis*							
**Study Name**	**Setting**	**Primary Study design**	**Population represented**	**N**	Neonatal mortality rate**	Infant mortality rate**	**% LBW**	**% preterm**	**% SGA**	**% facility delivery**	N* (analyzed cohort for this study)
**Brazil (1982) **[11]	Urban Pelotas city, Rio Grande do Sul, Southern BRAZIL	Longitudinal Birth Cohort Survey	Population based, all births in Pelotas hospitals	5,914	11	28	7	5	17	100	3,526
**Brazil (1993) **[12]	Urban Pelotas city, Rio Grande do Sul, Southern BRAZIL	Longitudinal Birth Cohort Survey	Population based, all births in Pelotas hospitals	5,279	7	14	9	10	19	100	3,057
**Brazil (2004) **[13]	Urban Pelotas city, Rio Grande do Sul, Southern BRAZIL	Longitudinal Birth Cohort Survey	Population based, all births in Pelotas hospitals	4,287	10	17	11	16	15	100	2,326
**Philippines (1983) **[15]	Urban Cebu, PHILLIPINES	Longitudinal Health-nutritional survey of infant feeding patterns	Population based, random cluster sample of census	3,080	14	36	11	18	25	34	2,423
**Zimbabwe (1997) **[14]	Urban Harare, ZIMBABWE	RCT of maternal-neonatal Vitamin A supplementation	Facility based recruitment, 14 maternity clinics and hospitals	14,110	12***	93	14	8	33	100	7,908

*Primparous babies were excluded from the analysis.

**Per 1000 live births

***Enrollment of newborns occurred up to 96 hours after birth, and the study may have missed neonatal deaths prior to enrollment.

### Exposure/independent variable

The independent variable was birth interval (the time between the previous and index live birth). Birth interval was categorized as <18 vs. 18-<24 vs. 24-<36 vs. 36-<60 (reference) vs. ≥60 months. The reference category was chosen in light of previous literature reporting a plateau in risk between roughly 36 and 60 months [2], and also to have a long interval category with high enough prevalence to account for its possible adverse effects. Cut-offs lower than 18 months could not be used because there were too few pregnancies with such short intervals.

### Outcomes/dependent variables

SGA was defined as below the 10^th^ percentile of a gender-specific reference distribution at each completed gestational week, using births in the US in 1991 [19]. We selected this reference population for comparability of our results to existing literature, as a large number of publications have used this reference population. Preterm was defined as below 37 completed weeks of gestation. We also created gestational age-SGA combination categories: term-SGA, preterm-appropriate-for-gestational-age (AGA), preterm-SGA, and term-AGA (reference). Neonatal mortality was defined as death within 28 days of life and infant mortality as death within the first 365 days of life.

### Analysis

For each birth cohort, logistic regression was performed with the birth outcome as the dependent variable and birth interval, parity, age, and other potential confounders included as independent variables. In each dataset, the available socioeconomic and maternal nutrition variables were placed in the multivariate model to calculate adjusted odds ratios (aOR). The covariates are listed in Supplemental Table 1 in Additional file 1. The aORs of birth interval with adverse neonatal outcomes were pooled across studies using meta-analysis techniques. We used the random effects DerSimonian-Laird pooled ORs and 95% CIs to address heterogeneity across studies. Nulliparous women were excluded, as they had no preceding birth interval. We used Stata 12.0 (Stata Corp.) for analysis.

## Results

### Distribution of exposure/outcomes: individual birth cohorts

**Figure 1:**
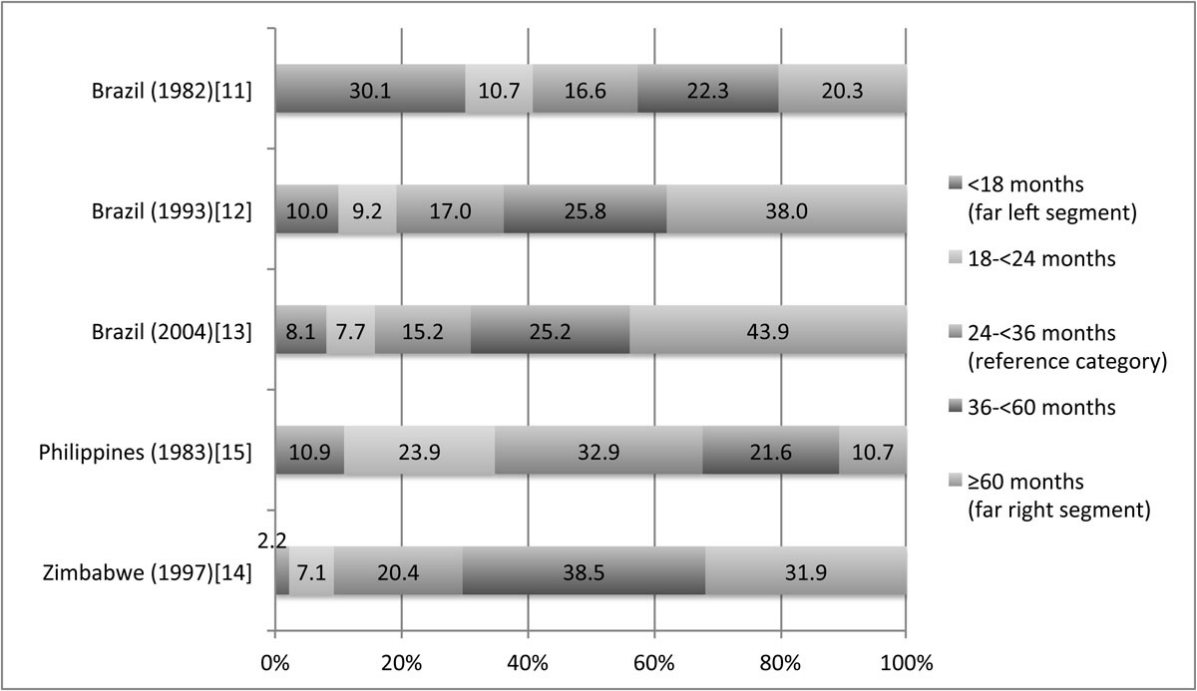
Percent of pregnancies within each birth interval exposure category.

The percentage of births in each birth interval category are shown in Figure 1. The 1982 Brazil study had the highest percentage of women in the <18 month birth interval category. The Philippines data was the most evenly distributed in terms of the percentage of women in the short versus reference versus long interval categories, and finally, the 1993 and 2004 Brazil and Zimbabwe studies had the highest percentage of mothers in the longer birth interval categories.

Table 2 shows the prevalence of the adverse neonatal outcomes for each study. The SGA prevalence ranged from 16.7-32.8% and preterm from 5.0-17.0%. NMR ranged from 9 to 21. The low NMR of 9 in Zimbabwe is most likely due to lower risk associated with facility deliveries in Harare and also enrollment into the study extending up to 96 hours after delivery; we expect the study missed some neonatal deaths by enrolling beyond time of delivery. IMR ranged from 19 to 78.

**Table 2:** Prevalence of adverse newborn outcomes in each study, among newborns included in the analysis

Study	SGA <10%	Preterm	Term-SGA	Preterm-AGA	Preterm-SGA	Neonatal Mortality Rate*	Infant mortality Rate*
**Brazil (1982) **[11]	21.1	5.0	12.4	3.9	1.0	21	40
**Brazil (1993) **[12]	20.3	10.2	15.4	9.3	1.0	16	24
**Brazil (2004) **[13]	16.7	16.1	11.8	14.3	1.7	11	19
**Philippines (1983) **[15]	25.3	17.0	22.7	14.4	2.6	13	33
**Zimbabwe (1997) **[14]	32.8	7.6	29.9	4.7	2.9	9**	78

*per 1000 live births

**Enrollment of newborns occurred up to 96 hours after birth, and the study may have missed neonatal deaths prior to enrollment.

SGA = small-for-gestational-age, AGA = appropriate-for-gestational-age. Preterm = below 37 completed weeks of gestation.

### Association of birth intervals and adverse outcomes

Supplemental Tables 2a-2c in Additional file 1 present the unadjusted and adjusted associations of birth interval length and adverse outcomes from each individual birth cohort and Table 3 presents the meta-analyzed pooled associations from the five studies.

**Table 3:** Meta-analyzed adjusted odds ratios of the association between short and long birth intervals and adverse neonatal and infant outcomes (36-<60 months as reference)

	**<18 months** **aOR (95% CI)**	**18-<24 months** **aOR (95% CI)**	**24-<36 months** **aOR (95% CI)**	**36-<60** **months**	**≥60 months** **aOR (95% CI)**
**SGA**	1.51 (1.31, 1.75)	1.23 (1.03, 1.48)	1.05 (0.87, 1.27)	Ref	1.22 (1.07, 1.39)
**Preterm**	1.58 (1.19, 2.10)	1.16 (0.94, 1.42)	1.02 (0.87, 1.19)	Ref	1.05 (0.88, 1.26)
Term-SGA*	1.39 (1.18, 1.64)	1.15 (0.98, 1.35)	1.01 (0.83, 1.22)	Ref	1.14 (1.03, 1.27)
Preterm-AGA*	1.45 (1.05, 1.99)	1.09 (0.86, 1.37)	1.01 (0.85, 1.21)	Ref	1.06 (0.87, 1.29)
Preterm-SGA*	3.04 (2.02, 4.58)	1.58 (1.01, 2.49)	0.92 (0.65, 1.31)	Ref	1.19 (0.87, 1.63)
**Neonatal mortality**	1.49 (0.93, 2.37)	1.07 (0.52, 2.22)	0.95 (0.62, 1.47)	Ref	1.01 (0.68, 1.49)
**Infant mortality**	1.83 (1.19, 2.81)	1.08 (0.66, 1.78)	1.17 (0.96, 1.43)	Ref	1.01 (0.84, 1.22)

*Reference: Term-AGA

### Short birth interval

**Figure 2:**
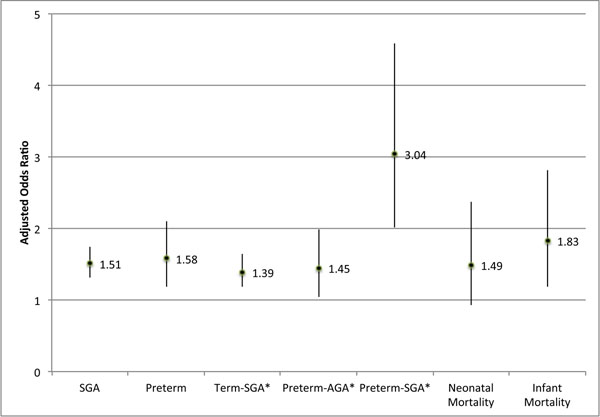
**Associations between birth interval <18 months (reference: 36-<60 months) and adverse neonatal and infant outcomes.** SGA = Small-for-gestational-age, below the 10^th^ percentile of a gender-specific reference distribution at each completed gestational week, using births in the US in 1991 [19]. AGA = Appropriate-for-gestational-age. Preterm = below 37 completed weeks of gestation. *Reference: term-AGA.

Birth interval <18 months was statistically significantly associated with almost all adverse neonatal outcomes. It had a pooled aOR of 1.51 (95% CI: 1.31, 1.75) for SGA and 1.58 (95% CI: 1.19, 2.10) for preterm. Examining the SGA and preterm combination outcomes, the short interval increased the odds of term-SGA (pooled aOR: 1.39, 95% CI: 1.18, 1.64) and preterm-AGA (pooled aOR: 1.45, 95% CI: 1.05, 1.99), and had a 3-fold increase in odds of preterm-SGA (pooled aOR: 3.04, 95% CI: 2.02, 4.58). See Figure 2 and Table 3.

Birth interval <18 months did not increase the risk of neonatal mortality to a level of statistical significance (aOR: 1.49, 95% CI: 0.93-2.37), however we observed an 83% increase in odds of infant mortality (1.83, 95% CI: 1.19, 2.81). See Figure 2 and Table 3. The 18-<24 month category was significantly associated with SGA (pooled aOR: 1.23, 95% CI: 1.03, 1.48), and although it had no significant association with preterm alone, the risk of the combined outcome of preterm-SGA was significantly increased by 58% (95% CI: 1.01, 2.49). None of the associations for the 24<36 month category were statistically significant, and were all close to 1. The increased risk of adverse outcomes associated with short birth interval appears to have a dose response relationship, as the magnitudes of the association are higher in the shorter birth interval categories.

### Long birth interval

Birth interval of ≥60 months was associated with a slight increase in odds of SGA (pooled aOR: 1.14, 95% CI 1.07-1.39) and term-SGA (pooled aOR: 1.14, 95% CI: 1.03, 1.27). The risks for other outcomes were small and non-significant.

## Discussion

In our meta-analysis, short birth interval (<18 months) was significantly associated with SGA (aOR 1.51), preterm (aOR 1.58), and infant mortality (aOR 1.83). We observed a dose response relationship, with the magnitude of risk increasing as the birth intervals got shorter from the reference 36-<60 month category. Birth interval <18 months carried a substantially higher (three-fold) risk of delivering an infant who is both preterm and SGA compared to those who had a reference birth interval; preterm-SGA babies carry substantially higher risk of mortality than those born term-AGA [10].

Our findings produced a similar magnitude of associations as previous literature for short intervals with SGA and preterm outcomes, although the results cannot be directly compared due to different birth interval cut-offs and definitions. Conde-Agudelo et al.’s meta-analysis found an adjusted odds ratio of 1.26 (95% CI: 1.18-1.33) for SGA and 1.40 (95% CI 1.24, 1.58) for preterm, examining an interpregnancy interval (IPI) (period between birth and conception) of <6 months, against a reference of 18 to 23 months [1]. However, the definition of SGA accepted for inclusion in the meta-analysis was not clearly defined. In Wendt et al.’s meta-analysis [20], preterm associations had similar magnitudes also looking at an IPI of six months with a range of reference intervals, but they did not examine SGA or IUGR because of the inconsistencies in definitions across studies. Using IPI <6 month exposure and 18-<24 month reference, a study examining 173,205 children from Utah birth records (1989-1996) saw an SGA aOR of 1.3 (95% CI: 1.2-1.4) and a preterm aOR of 1.4 (95% CI: 1.3-1.5) [7]. A separate study used Michigan birth records and linked births by mother to create longitudinal cohorts; that study noted statically significant aORs with low birthweight, ranging from 1.2 to 1.5 depending on birth order of the children [21]. However, the low birthweight outcome is not directly comparable to SGA or preterm.

In our meta-analysis, short birth interval was significantly associated with increased infant mortality risk, however had no significant association with neonatal mortality risk. This finding may be driven by the smaller number of neonatal deaths, compared to infant deaths; we noticed increased risk in all datasets, but confidence intervals were wide and crossed unity in the pooled association. Incomplete neonatal mortality information in the Zimbabwe dataset may also have affected the association. Another possible explanation may be the confounding effect of breastfeeding. Those who fail to breastfeed will regain their fecundity sooner than those who do, leading to shorter birth intervals. We also expect mothers to repeat negative breastfeeding patterns for the subsequent child [22], which impacts the child’s survival in the infant period. Therefore it may not be the physiological effect of short birth intervals, but breastfeeding practices correlated with short intervals that lead to adverse infant outcomes. We did not have relevant information available to explore this hypothesis. A meta-analysis using 17 DHS datasets found neonatal and infant mortality associations with birth interval <18 months stronger than what we found in our data (neonatal: aOR 2.72, 95% CI 2.3-3.2, infant: aOR 2.84, 95% CI 2.5-3.2); however the study used cross-sectional data and a reference category of 36-47 months [2].

Long intervals do not appear to have a strong adverse association with neonatal outcomes; we only observed a statistically significant 12% increase in odds in SGA but no association with any other adverse outcomes. A meta-analysis of DHS data showed no adverse association for long birth intervals as well [2]. In contrast, Conde-Agudelo et al’s meta-analysis found 36% increased odds of SGA (95% CI: 1.20, 1.54) and 27% increased odds of preterm (95% CI: 1.17, 1.39) for IPI over 60 months (birth interval of approximately 69 months), but had a different reference category [1].

Some researchers have hypothesized that maternal depletion drives the association between short birth intervals and adverse neonatal outcomes; a mother may not have nutritionally and physiologically recovered enough before conceiving the next child. Upon controlling for available maternal nutritional variables, we witnessed no significant change in the magnitude of the associations. This may imply that nutritional depletion either plays no or a small role. A systematic review also found weak evidence to support the maternal depletion hypothesis [4], examining 15 studies that used anthropometric outcomes, maternal anemia or iron deficiency, and micronutrient deficiency as indicators of depletion. However, a separate study noted that only short birth interval children of higher birth order had a high risk of death [23]. It may be that nutritional depletion only plays a role following a cumulative effect of having multiple children or multiple short interval children. The same study also revealed fundamental background differences between mothers who completed their reproductive period with high fertility versus low fertility, and that low birth orders of high fertility mothers are worse off after a short interval than low birth orders of low fertility mothers after a short interval. Mothers who have low completed fertility may have background characteristics (i.e. better socioeconomic status) that allow them to tolerate nutritional and economic demands of short interval births, while high completed fertility mothers, who start worse off than low completed fertility mothers, may not have the capacity to handle those demands. Parity may be modifying the effect of birth intervals only when certain socioeconomic and/or nutritional conditions are present. The prospective cohort studies presented in our meta-analyses do not have information like mother’s final fertility that may serve as a proxy for effect modifiers or residual confounders that are not captured by available variables; the findings in the aforementioned study [23] suggests that we may have failed to address either some effect modification and/or confounding in our analysis.

Numerous other hypotheses on mechanisms linking birth intervals to adverse health outcomes have been identified [4]. Mechanisms with more evidence base include folate deficiency. While there is substantial evidence reporting folate deficiency following pregnancy, very few report on likelihood of folate deficiency among short birth interval mothers, and on the association between short birth interval and birth outcome among mothers who were not supplemented with folic acid. Regarding sibling competition theory, evidence implied that competition was not a major factor linking short birth intervals to neonatal mortality, but possibly for post-neonatal mortality. Other hypotheses include cervical inefficiency and vertical transmission of infections, but there is no clear evidence that supports these hypotheses.

The strength of our analysis is the use of high quality exposure, outcome, and confounder data from prospective birth cohorts. Unlike some cross-sectional survey data, the outcome information is collected soon before or around the time of birth. Also, by standardizing the categorization of birth intervals and outcomes, we were able to meta-analyze five studies with the same exposure and outcome definitions. One of the largest methodological issues with other meta-analyses is the heterogeneity of birth interval categorization, definition of SGA, use of birth-to-birth intervals versus birth-to-conception intervals (or IPI), and other exposure and/or outcome definitions.

The main weakness of this study and of almost all other studies reporting on birth interval is the inability to examine the associations taking into account the length of each component of a birth interval (birth to fecundity, fecundity to conception, and conception to birth.) Non-live birth outcomes (abortion, miscarriage, stillbirth) in between two live births may attenuate the association between short intervals and adverse outcomes by attributing more adverse outcomes to the reference or long birth interval. A study conducted in Bangladesh [24] noted differences in associations between an IPI <6 months with induced abortion, miscarriage, and stillbirth, depending on what outcome the IPI began with. The magnitude of association was highest among IPIs starting with live births for outcomes of induced abortion and miscarriage, but with stillbirths for outcome of stillbirth, although not all associations were statistically significantly different from each other. Furthermore, the first live births that we excluded from our analysis may contribute more information, as these children may have followed a pregnancy that ended in a non-live birth. The length of gestation would also affect the conception-to-birth period, and preterm outcomes could have a variety of etiologies that may not be captured through available confounding variables. We did not have complete pregnancy histories that would help us better explore these issues. Future research on birth intervals would benefit greatly from collecting appropriate data to distinguish these birth interval components and their predictors. Finally, there may have been residual confounders that were not fully captured in the available data, such as the mother’s history of preterm births and breastfeeding practices, as we only controlled for available nutritional and socioeconomic variables.

The associations we see between short birth intervals and adverse outcomes emphasize the importance of family planning interventions and the timing of the interventions. As Conde-Agudelo et al. have also stated [1], unmet need for family planning is not only a socioeconomic issue, but a public health issue for both the mother and the child. Assuming a 10% prevalence of short birth interval (<18 months) and infant mortality aOR of 1.83, lengthening the birth intervals of those individuals to ≥18 months could reduce infant mortality by 7.7%, a magnitude that is of public health significance. Furthermore, if a differential impact of short birth intervals exists by mothers’ background characteristics, unmet need poses a major health equity problem. While modern contraceptive use among women of reproductive age is ~70% in North America and Western Europe, it is only ~15% in Sub-Saharan Africa, with many countries reporting single digit figures [25]. Within countries also, there are huge equity gaps; taking Burkina Faso and Mozambique as examples, they have a close to a 30 percentage point difference in modern contraceptive use between the lowest wealth quintile (6.3% in Burkina Faso [26], 3.9% in Mozambique [27]) and the highest wealth quintile (35.5%, 34.8%). Equitable access to family planning interventions need to consciously target the most vulnerable women, as they may carry the highest health risks associated with short intervals and are also the least likely to have access to health education, contraceptives, and medical care.

## Conclusion

Our results suggest that birth intervals under 18 months are associated with adverse neonatal outcomes, with as high as an 83% increase in odds of infant mortality. Policymakers have the responsibility to secure family planning access to all women, which would benefit the health and the economy of the population. Programs and policies also need to focus on vulnerable mothers, as they may have higher risks associated with short intervals and are least likely to have interventions reach them. Finally, more operations research needs to be conducted to determine the most effective ways to delay subsequent births in LMICs.

In *LiST*, we recommend the inclusion of the associations between short birth intervals and adverse outcomes, with the understanding that birth intervals may have differential impact on neonatal and infant outcomes, depending on the baseline condition of the mother.

## Competing interests

We report no competing interests.

## Authors’ contributions

NK was responsible for study design, data collection, literature review, analyses of primary datasets and the meta-analyses, interpretation of results, and writing of the manuscript. ACL and JK were responsible for study design, data collection, interpretation of results, and helped draft the manuscript. MFS analyzed the primary datasets and reviewed the manuscript. CV, LA, JH, RN contributed data and reviewed the manuscript. REB contributed to study design and interpretation of results, and reviewed the manuscript. All authors read and approved the final manuscript.

## Supplementary Material

Additional file 1 Supplemental material. Click here for file

## Declarations

The publication costs for this supplement were funded by a grant from the Bill & Melinda Gates Foundation to the US Fund for UNICEF (grant 43386 to "Promote evidence-based decision making in designing maternal, neonatal, and child health interventions in low- and middle-income countries”). The Supplement Editor is the principle investigator and lead in the development of the Lives Saved Tool (LiST), supported by grant 43386. He declares that he has no competing interests.

This article has been published as part of *BMC Public Health* Volume 13 Supplement 3, 2013: The Lives Saved Tool in 2013: new capabilities and applications. The full contents of the supplement are available online at http://www.biomedcentral.com/bmcpublichealth/supplements/13/S3.
