# Supplementary material for: The associations of birth intervals with small-for-gestational-age, preterm, and neonatal and infant mortality: a meta-analysis
Source: BMC Public Health. 2013 Sep 17;13(Suppl 3):S3. doi: 10.1186/1471-2458-13-S3-S3 (PMC3847557; doi:10.1186/1471-2458-13-S3-S3)
Supplement: Additional file 1 — Supplemental material. [file 1471-2458-13-S3-S3-S1.pdf]

Kozuki N, et al. The associations of birth intervals with small-for-gestational-age, preterm, and neonatal and infant mortality: a meta-analysis.

Supplemental Table 1: Adjustment variables used in the adjusted logistic regression analyses, by study

Country	Socioeconomic adjustment variables	Maternal nutrition adjustment variables	Reproductive health variables
**Brazil (1982)[11]**	Income, maternal education	Height, BMI	Maternal age, parity
**Brazil (1993)[12]**	Income, maternal education	Height, BMI	Maternal age, parity
**Brazil (2004)[13]**	Income, maternal education	Height, BMI	Maternal age, parity
**Philippines (1983)[15]**	Land ownership, literacy, housing structure	Height, BMI, MUAC	Maternal age, parity
**Zimbabwe (1997)[14]**	Income quintiles	Postpartum MUAC	Maternal age, parity

Kozuki N, et al. The associations of birth intervals with small-for-gestational-age, preterm, and neonatal and infant mortality: a meta-analysis.

Supplemental Table 2a: Unadjusted and adjusted associations between birth intervals and adverse newborn outcomes, by study

		SGA (Reference: AGA)						Preterm (Reference: Term)					
Study name	Birth interval	Unadjusted			Adjusted			Unadjusted			Adjusted		
		OR	LI	UI	OR	LI	UI	OR	LI	UI	OR	LI	UI
**Brazil (1982)[11]**	<18	2.0	1.6	2.6	1.9	1.4	2.5	1.2	0.8	1.9	1.3	0.8	2.0
	18-<24	1.7	1.2	2.3	1.6	1.1	2.3	1.1	0.6	1.9	1.0	0.5	1.8
	24-<36	1.4	1.0	1.8	1.3	0.9	1.8	1.0	0.6	1.6	0.9	0.5	1.6
	36-<60	Ref	Ref	Ref	Ref	Ref	Ref	Ref	Ref	Ref	Ref	Ref	Ref
	≥60	1.3	1.0	1.7	1.5	1.1	2.0	1.0	0.6	1.6	0.9	0.5	1.6
**Brazil (1993)[12]**	<18	1.5	1.1	2.1	1.4	1.0	2.1	2.0	1.3	3.0	1.9	1.2	3.0
	18-<24	1.4	1.0	2.0	1.5	1.0	2.1	0.9	0.6	1.6	1.0	0.6	1.7
	24-<36	1.3	1.0	1.8	1.3	0.9	1.7	1.4	1.0	2.1	1.3	0.9	1.9
	36-<60	Ref	Ref	Ref	Ref	Ref	Ref	Ref	Ref	Ref	Ref	Ref	Ref
	≥60	1.1	0.9	1.4	1.3	1.0	1.6	1.3	0.9	1.7	1.3	0.9	1.7
**Brazil (2004)[13]**	<18	1.6	1.1	2.5	1.2	0.6	2.3	2.3	1.5	3.5	2.1	1.2	3.6
	18-<24	1.0	0.6	1.7	0.8	0.4	1.7	1.6	1.1	2.5	1.5	0.8	2.7
	24-<36	1.0	0.7	1.5	0.9	0.5	1.6	1.0	0.7	1.5	1.0	0.6	1.7
	36-<60	Ref	Ref	Ref	Ref	Ref	Ref	Ref	Ref	Ref	Ref	Ref	Ref
	≥60	1.1	0.9	1.5	1.4	0.9	2.0	0.7	0.5	1.0	0.8	0.6	1.2
**Philippines (1983)[15]**	<18	1.28	0.92	1.78	1.31	0.93	1.83	0.92	1.78	1.31	0.93	1.83	1.05
	18-<24	1.07	0.79	1.46	1.13	0.82	1.56	0.79	1.46	1.13	0.82	1.56	0.75
	24-<36	0.76	0.57	1.01	0.76	0.57	1.02	0.57	1.01	0.76	0.57	1.02	0.59
	36-<60	Ref	Ref	Ref	Ref	Ref	Ref	Ref	Ref	Ref	Ref	Ref	Ref
	≥60	0.84	0.58	1.23	0.94	0.64	1.40	0.58	1.23	0.94	0.64	1.4	0.61
**Zimbabwe (1997)[14]**	<18	1.47	1.15	1.88	1.48	1.15	1.90	1.15	1.88	1.48	1.15	1.9	1.35
	18-<24	1.17	0.94	1.46	1.11	0.89	1.38	0.94	1.46	1.11	0.89	1.38	1.37
	24-<36	1.08	0.94	1.24	1.06	0.93	1.21	0.94	1.24	1.06	0.93	1.21	1.13
	36-<60	Ref	Ref	Ref	Ref	Ref	Ref	Ref	Ref	Ref	Ref	Ref	Ref
	≥60	1.12	0.99	1.26	1.13	1.00	1.28	0.99	1.26	1.13	1.00	1.28	1.02

Kozuki N, et al. The associations of birth intervals with small-for-gestational-age, preterm, and neonatal and infant mortality: a meta-analysis.

SGA = small-for-gestational-age, defined as below the 10th percentile of the U.S. 1991 reference distribution described by Alexander and colleagues [19]. AGA = appropriate-for-gestational-age. Preterm = below 37 completed weeks of gestation

Kozuki N, et al. The associations of birth intervals with small-for-gestational-age, preterm, and neonatal and infant mortality: a meta-analysis.

Supplemental Table 2b: Unadjusted and adjusted associations between birth intervals and adverse newborn outcomes, by study

		Term-SGA (Reference: Term-AGA)						Preterm-AGA (Reference: Term-AGA)						Preterm-SGA (Reference: Term-AGA)					
Study name	Birth interval	Unadjusted			Adjusted			Unadjusted			Adjusted			Unadjusted			Adjusted		
		OR	LI	UI	OR	LI	UI	OR	LI	UI	OR	LI	UI	OR	LI	UI	OR	LI	UI
**Brazil (1982) [11]**	<18	1.6	1.2	2.2	1.6	1.1	2.3	1.1	0.7	1.7	1.1	0.7	1.9	3.5	1.2	10.8	3.7	1.0	13.4
	18-<24	1.4	0.9	2.1	1.4	0.9	2.1	0.8	0.4	1.6	0.7	0.5	1.5	4.0	1.2	13.8	3.6	0.8	15.7
	24-<36	1.2	0.8	1.7	1.3	0.8	1.8	1.0	0.6	1.6	0.9	0.5	1.6	1.4	0.3	5.7	1.4	0.3	6.3
	36-<60	Ref	Ref	Ref	Ref	Ref	Ref	Ref	Ref	Ref	Ref	Ref	Ref	Ref	Ref	Ref	Ref	Ref	Ref
	≥60	0.9	0.6	1.2	1.0	0.7	1.6	0.8	0.5	1.4	0.8	0.6	1.5	1.9	0.6	6.6	2.2	0.5	8.5
**Brazil (1993) [12]**	<18	1.6	1.1	2.3	1.6	1.1	2.5	2.2	1.4	3.3	2.1	1.3	3.4	1.2	0.1	11.3	1.6	0.1	19.0
	18-<24	1.4	0.9	2.0	1.4	0.9	2.1	0.9	0.5	1.5	1.0	0.6	1.7	3.1	0.6	15.7	3.5	0.6	22.5
	24-<36	1.3	0.9	1.8	1.3	0.9	1.8	1.5	1.0	2.2	1.3	0.9	2.0	1.7	0.3	8.6	1.6	0.2	11.7
	36-<60	Ref	Ref	Ref	Ref	Ref	Ref	Ref	Ref	Ref	Ref	Ref	Ref	Ref	Ref	Ref	Ref	Ref	Ref
	≥60	1.2	0.9	1.5	1.3	1.0	1.7	1.3	0.9	1.8	1.2	0.9	1.7	1.9	0.5	7.1	3.6	0.7	17.5
**Brazil (2004) [13]**	<18	1.5	0.9	2.7	1.0	0.5	2.4	2.6	1.7	4.0	2.1	1.2	3.7	3.7	0.5	8.1	1.9	0.3	10.5
	18-<24	1.0	0.5	1.8	0.7	0.3	1.7	1.8	1.1	2.8	1.6	0.9	2.8	1.1	0.1	4.8	0.6	0.1	5.7
	24-<36	0.9	0.5	1.4	0.7	0.4	1.3	1.0	0.7	1.5	1.1	0.6	1.8	1.7	0.3	3.3	0.7	0.1	3.5
	36-<60	Ref	Ref	Ref	Ref	Ref	Ref	Ref	Ref	Ref	Ref	Ref	Ref	Ref	Ref	Ref	Ref	Ref	Ref
	≥60	1.0	0.7	1.4	1.2	1.0	1.8	0.7	0.5	1.0	0.8	0.6	1.4	1.8	0.4	2.5	0.9	0.3	3.1
**Philippines (1983) [15]**	<18	1.19	0.84	1.68	1.26	0.88	1.80	0.89	0.58	1.38	0.98	0.63	1.19	1.26	0.84	1.68	0.89	0.88	1.80
	18-<24	1.03	0.74	1.43	1.12	0.80	1.57	1.00	0.68	1.47	1.03	0.73	1.08	1.12	0.74	1.43	1.00	0.80	1.57
	24-<36	0.75	0.56	1.02	0.76	0.56	1.03	1.02	0.73	1.43	0.75	0.75	1.06	0.76	0.56	1.02	1.02	0.56	1.03
	36-<60	Ref	Ref	Ref	Ref	Ref	Ref	Ref	Ref	Ref	Ref	Ref	Ref	Ref	Ref	Ref	Ref	Ref	Ref
	≥60	0.82	0.55	1.23	0.92	0.61	1.40	0.87	0.55	1.39	0.82	0.55	0.88	0.92	0.55	1.23	0.87	0.61	1.40
**Zimbabwe (1997) [14]**	<18	1.31	1.00	1.72	1.31	1.00	1.72	1.47	0.87	2.50	1.31	0.80	1.39	1.31	1.00	1.72	1.47	1.00	1.72
	18-<24	1.15	0.92	1.46	1.09	0.86	1.37	1.21	0.76	1.94	1.13	0.70	1.15	1.09	0.92	1.46	1.21	0.86	1.37
	24-<36	1.08	0.94	1.24	1.06	0.92	1.22	0.85	0.62	1.17	0.84	0.61	1.08	1.06	0.94	1.24	0.92	0.85	1.22

	36-<60	Ref	Ref	Ref	Ref	Ref	Ref	Ref	Ref	Ref	Ref	Ref	Ref	Ref	Ref	Ref	Ref	Ref	Ref
	≥60	1.14	1.01	1.29	1.15	1.02	1.31	1.25	0.97	1.60	1.27	0.98	1.14	1.15	1.01	1.29	1.25	1.02	1.31

SGA = small-for-gestational-age, defined as below the 10th percentile of the U.S. 1991 reference distribution described by Alexander and colleagues [19]. AGA = appropriate-for-gestational-age. Preterm = below 37 completed weeks of gestation

Supplemental Table 2c: Unadjusted and adjusted associations between birth intervals and risk of neonatal and infant mortality, by study

		Neonatal Mortality						Infant mortality					
Study name	birth interval	unadj			adj			unadj			adj		
		OR	LI	UI	OR	LI	UI	OR	LI	UI	OR	LI	UI
**Brazil (1982) [11]**	<18	1.5	0.8	2.9	1.2	0.6	2.6	3.0	1.7	5.3	1.7	0.9	3.2
	18-<24	1.4	0.6	3.4	1.3	0.5	3.4	2.1	1.1	4.3	2.0	0.9	4.2
	24-<36	1.3	0.6	2.9	1.0	0.4	2.5	2.1	1.1	4.1	1.7	0.8	3.4
	36-<60	Ref	Ref	Ref	Ref	Ref	Ref	Ref	Ref	Ref	Ref	Ref	Ref
	≥60	1.2	0.6	2.5	0.9	0.4	2.1	1.5	0.8	2.8	1.0	0.5	2.1
**Brazil (1993) [12]**	<18	2.6	1.1	6.1	1.9	0.8	4.8	1.9	0.9	3.9	1.5	0.7	3.2
	18-<24	0.5	0.1	2.3	0.5	0.1	2.1	0.8	0.3	2.1	0.5	0.2	1.6
	24-<36	1.0	0.4	2.5	0.8	0.3	2.1	1.2	0.6	2.4	1.0	0.5	2.2
	36-<60	Ref	Ref	Ref	Ref	Ref	Ref	Ref	Ref	Ref	Ref	Ref	Ref
	≥60	1.1	0.5	2.4	1.0	0.4	2.1	0.9	0.5	1.6	0.8	0.4	1.5
**Brazil (2004) [13]**	<18	1.0	0.2	5.2	0.6	0.1	5.3	0.6	0.1	2.9	0.3	0.1	2.7
	18-<24	1.1	0.2	5.5	small n			1.3	0.4	4.3	0.3	0.1	2.7
	24-<36	1.1	0.3	3.9	0.9	0.2	4.7	1.5	0.6	3.7	0.6	0.2	2.5
	36-<60	Ref	Ref	Ref	Ref	Ref	Ref	Ref	Ref	Ref	Ref	Ref	Ref
	≥60	1.1	0.4	2.9	1.2	0.3	4.3	1.2	0.5	2.5	1.1	0.4	3.1
**Philippines (1983) [15]**	<18	3.13	0.78	12.59	3.50*	0.86	14.33	2.07	0.99	4.32	2.65	0.78	3.13
	18-<24	2.58	0.66	10.05	2.79*	0.71	10.95	1.49	0.72	3.11	1.59	0.66	2.58
	24-<36	1.53	0.39	5.95	1.49*	0.38	5.82	1.02	0.50	2.06	1.12	0.39	1.53
	36-<60	Ref	Ref	Ref	Ref	Ref	Ref	Ref	Ref	Ref	Ref	Ref	Ref
	≥60	2.05	0.41	10.21	1.96*	0.38	5.82	0.74	0.26	2.10	0.79	0.41	2.05
**Zimbabwe (1997) [14]**	<18	1.22	0.37	4.07	1.26	0.38	4.20	2.34	1.65	3.34	1.22	0.37	2.43
	18-<24	0.55	0.13	2.34	0.54	0.13	2.29	1.12	0.77	1.62	0.55	0.13	1.10
	24-<36	0.91	0.45	1.81	0.91	0.45	1.81	1.15	0.92	1.45	0.91	0.45	1.17
	36-<60	Ref	Ref	Ref	Ref	Ref	Ref	Ref	Ref	Ref	Ref	Ref	Ref
	≥60	0.82	0.44	1.53	0.90	0.48	1.68	0.98	0.79	1.20	0.82	0.44	1.04

*Height removed from this set of analyses due to convergence issues.
